# Emotional resiliency and life satisfaction among teachers of Chinese as a foreign language: mediating chain model with grit and employability and gender moderation

**DOI:** 10.3389/fpsyg.2025.1450617

**Published:** 2025-02-26

**Authors:** Yicun Jia

**Affiliations:** International School, International Students Center, Beijing Youth Politics College, Beijing, China

**Keywords:** Chinese as a foreign language (CFL) teachers, emotional resilience, grit, employability, life satisfaction

## Abstract

**Objective:**

To investigate the relationships between emotional resilience, grit, employability, and life satisfaction among Chinese as a Foreign Language (CFL) teachers in China, with a focus on gender differences.

**Methods:**

A sample of 1,003 CFL teachers participated in a survey assessing emotional resilience, grit, employability, and life satisfaction. Data were analyzed using descriptive statistics, correlation analyses, and mediation and moderation models with the PROCESS macro.

**Results:**

Emotional resilience did not have a significant direct effect on life satisfaction but showed substantial indirect effects through grit and employability. Emotional resilience positively influenced grit, which in turn enhanced employability and increased life satisfaction. Gender differences were observed grit was a more critical mediator for males, while employability was a stronger mediator for females.

**Conclusion:**

The study highlights the importance of grit and employability in enhancing life satisfaction among CFL teachers. Gender-specific interventions to bolster these mediating factors are recommended to support the wellbeing and professional success of CFL teachers.

**Implications:**

Educational institutions should develop professional development programs and support systems that foster emotional resilience, grit, and employability, with attention to gender-specific needs, to improve job satisfaction and effectiveness among CFL teachers.

## Introduction

Teaching Chinese as a Foreign Language (CFL) has gained significant importance due to China’s expanding global influence and the strategic role of Mandarin in international communication (Hsiang et al., 2023; Xu and Stahl, 2023). As key facilitators of linguistic and cultural exchange, CFL teachers play a critical role in promoting language acquisition and fostering cross-cultural understanding (Liang and Apedoe, 2025). However, despite the rising demand for CFL programs globally, many CFL teachers face substantial challenges, including professional stress, role overload, and limited support networks (Wang H. et al., 2023).

Existing research has explored some of these challenges, focusing on general stress factors, gender dynamics, and teaching effectiveness in various contexts. However, few studies have investigated how psychological traits such as grit and employability interact with emotional resilience to influence teachers’ life satisfaction, particularly in the case of CFL teachers operating in diverse cultural environments (Fu, 2025; Yang et al., 2025). Moreover, the literature often neglects how gender-specific factors mediate these relationships, leaving a critical gap in understanding the unique experiences of female teachers.

This study aims to fill these gaps by examining the mediating roles of grit and employability in the relationship between emotional resilience and life satisfaction among CFL teachers. The novelty of this research lies in its emphasis on gender differences, providing a more nuanced understanding of how male and female teachers navigate professional challenges. By addressing this overlooked dimension, the study contributes to the development of targeted support strategies to enhance teachers’ wellbeing and career satisfaction, ultimately improving the quality of CFL instruction.

## Theoretical framework and hypotheses development

### Teaching Chinese as a foreign language (CFL) in China: a gender perspective

Teaching CFL has garnered significant attention in recent years, reflecting China’s growing global influence and the increasing importance of the Chinese language in international communication (Chan et al., 2022; Jiang, 2025). The role of CFL teachers is pivotal in this cultural and linguistic exchange, as they serve as the primary facilitators of language acquisition for non-native speakers. These educators not only impart linguistic skills but also play a crucial role in promoting Chinese culture and fostering cross-cultural understanding, as well as in supporting international trade and economic development (Xu, 2024).

The relevance of CFL has surged alongside China’s economic ascendancy and its expanding geopolitical footprint. Mandarin Chinese, as the most spoken language globally, has become a strategic asset, prompting a rise in demand for CFL programs both domestically and internationally (Smith, 2023). Institutions ranging from primary schools to universities have incorporated Chinese language courses into their curricula, while international schools and Confucius Institutes worldwide have bolstered this trend. Consequently, CFL teachers are increasingly sought after, with their expertise becoming a critical component in educational and professional arenas (Wang, 2023).

Despite the growing demand and recognition, CFL teachers face a myriad of challenges that contribute to professional stress and difficulties. One of the primary stressors is the high expectation placed on them to deliver effective language instruction while simultaneously promoting cultural awareness. The dual responsibility of language teaching and cultural ambassadorship can be overwhelming, especially for those working in multicultural and multilingual environments (Zhang, 2025).

Another significant challenge is the need for constant adaptation to diverse learner needs. CFL teachers often encounter students with varying levels of language proficiency, learning styles, and cultural backgrounds (Ma et al., 2017). This diversity requires teachers to employ a wide range of pedagogical strategies and resources, which can be demanding and time-consuming. Moreover, the lack of standardized teaching materials and methodologies for CFL can exacerbate this issue, forcing educators to develop customized curricula and teaching aids (Hsiang et al., 2023).

Gender plays a crucial role in the stress and difficulties experienced by CFL teachers (Xiao and Asadullah, 2020). Female educators, in particular, often face unique challenges that can compound their professional stress. Studies indicate that women in teaching positions are more likely to experience role overload, balancing their professional responsibilities with societal expectations related to family and caregiving roles. This dual burden can lead to higher levels of stress and burnout among female CFL teachers (Zhou, 2023).

Additionally, gender-based expectations and biases can influence the professional experiences of CFL teachers. Female teachers may encounter gender stereotypes that undermine their authority and professional competence, further exacerbating their stress levels (Peng et al., 2023). They might also have fewer opportunities for career advancement and professional development compared to their male counterparts, contributing to job dissatisfaction and feelings of isolation (Horta and Tang, 2023).

CFL teachers frequently experience professional isolation, particularly those working in regions with limited support networks or access to professional development opportunities. This isolation can lead to feelings of burnout and decreased job satisfaction. Furthermore, the administrative burdens associated with their roles, such as curriculum planning, student assessment, and institutional reporting, add to their workload and stress levels (Ogbari, 2024).

The rapid technological advancements and the integration of digital tools in language teaching also present both opportunities and challenges for CFL educators. While technology can enhance language learning experiences, it requires teachers to continuously update their digital literacy skills and adapt to new teaching platforms and tools, which can be a source of additional pressure (Hoque and Atheef, 2024).

Hence, while the role of CFL teachers is crucial and increasingly relevant in today’s globalized world, the stress and difficulties they face, particularly from a gender perspective, cannot be overlooked. Addressing these challenges through research specific focused on their professional development could help to ensure the wellbeing and effectiveness of CFL educators.

## Emotional resilience as teachers’ capability and employability

Emotional resilience is defined as the ability to adapt to, recover from, and thrive in the face of adversity and stress. For teachers of Chinese as a Foreign Language (CFL), this capability is crucial as it allows them to manage the emotional demands of teaching, maintain a positive outlook, and sustain their passion for educating diverse student populations. Resilient CFL teachers can effectively handle classroom challenges, maintain constructive relationships with students and colleagues, and bounce back from setbacks (Smaliukienè et al., 2024).

Employability refers to the set of skills, knowledge, and attributes that make an individual desirable to employers and capable of securing and maintaining employment. For CFL teachers, employability encompasses not only pedagogical skills and subject matter expertise but also emotional resilience. Teachers with high employability can adapt to changing educational environments, incorporate new teaching methods, and continually develop professionally. Emotional resilience significantly enhances employability by enabling teachers to cope with job-related stress, embrace new challenges, and demonstrate reliability and stability in their roles (Bagdžiūnienė et al., 2023).

The interrelationship between emotional resilience and employability is evident in the teaching profession. Teachers who possess emotional resilience are better equipped to navigate the complexities of their roles, which enhances their employability. This resilience allows them to manage stress effectively, maintain high performance, and remain committed to their professional growth. Consequently, they become more attractive candidates for employment and are better positioned to advance in their careers (Lin and Felipe, 2023).

Teaching CFL presents unique challenges that test teachers’ emotional resilience and employability. High proficiency in Mandarin and deep cultural knowledge require continuous learning and adaptation. Creating engaging and effective learning experiences for students with different language backgrounds and learning styles demands innovation and flexibility, which can be mentally and emotionally taxing. Additionally, the pressure to achieve high educational outcomes and meet expectations from students and institutions contributes to considerable stress (Wang et al., 2024).

Investing in empirical research on emotional resilience is crucial for enhancing CFL teachers’ capabilities and employability. Hence, the present study that focus on understanding the factors contributing to CFL teachers’ Wellbeing through mediational variables could improve teachers’ employability and impact, in turn, their Wellbeing. The final aim is identifying effective practices and interventions through empirical research, that educational institutions can apply to support their staff in achieving long-term career success and job satisfaction (Smaliukienè et al., 2024).

## Mediating role of grit and employability in predicting life satisfaction

Grit, defined as perseverance and passion for long-term goals, is a significant psychological trait that influences both employability and life satisfaction. For teachers of Chinese as a Foreign Language (CFL), grit is essential in navigating the demanding and evolving educational landscape (Fu, 2025). This perseverance helps them continually develop professionally and personally, leading to higher employability and job satisfaction (Cayupe et al., 2023).

For CFL teachers, key employability attributes include career agility, cultural ingenuity, and proactive career resilience. Career agility involves adapting to new opportunities and challenges in the educational field. Cultural ingenuity refers to the ability to effectively interact with and teach students from diverse cultural backgrounds. Proactive career resilience is the capacity to remain confident and proactive in career development despite setbacks (Ismail et al., 2023).

Grit enhances employability by fostering self-regulatory behaviors such as continuous learning and adaptation. Teachers with high levels of grit are more likely to persist in improving their skills and competencies, thus meeting the evolving demands of their profession. This persistent effort not only makes them more employable but also contributes to their overall life satisfaction by achieving long-term career goals and personal fulfillment (Cabaron and Oco, 2023).

Life satisfaction, as a relevant Wellbeing indicator, is significantly influenced by the successful integration of grit and employability. Teachers who demonstrate high levels of grit tend to navigate professional challenges more effectively, leading to greater job stability and satisfaction. This stability and sense of achievement in their professional lives translate into higher overall life satisfaction. The ability to overcome obstacles and achieve career goals through perseverance directly impacts their sense of purpose and wellbeing, along all the lifetime (Hu et al., 2024).

## Gender moderation of the indirect effects of emotional resilience on teachers’ wellbeing through grit and employability

Emotional resilience is crucial for teachers, enabling them to manage the complex demands of their profession. Grit, characterized by perseverance and sustained passion for long-term goals, serves as a vital mediating factor between emotional resilience and life satisfaction. Teachers with high emotional resilience are likely to exhibit higher levels of grit, which in turn enhances their life satisfaction. This indirect effect suggests that resilience fosters a persistent and passionate approach to teaching, leading to greater personal and professional fulfillment (Simonton et al., 2023).

Employability, encompassing skills, knowledge, and personal attributes that facilitate securing and maintaining employment, is another mediator in the relationship between emotional resilience and life satisfaction. Teachers with high emotional resilience are better equipped to develop and sustain employability attributes such as adaptability, continuous learning, and effective interpersonal skills. This enhancement in employability contributes to greater job stability and career satisfaction, ultimately leading to higher overall life satisfaction. Emotional resilience thus indirectly boosts life satisfaction by improving teachers’ employability and their ability to thrive in diverse educational settings (Yang et al., 2025).

The combined effect of grit and employability further elucidates the pathway from emotional resilience to life satisfaction. Emotional resilience enhances grit, which subsequently improves employability by fostering a commitment to long-term professional development and goal attainment. This chain mediation effect highlights how emotional resilience indirectly contributes to life satisfaction through a sequential improvement in both grit and employability. Teachers who are resilient are likely to persist through challenges (grit), enhance their career-related skills and opportunities (employability), and thereby achieve higher levels of life satisfaction (Shao, 2023).

Gender stereotypes significantly influence the moderation of these indirect effects. Males often benefit from societal perceptions that align with traits such as perseverance and goal-oriented behavior, which are associated with grit. The magnetic male stereotype suggests that men who demonstrate context-specific goal-setting mindsets are viewed positively, enhancing their professional and personal outcomes. This societal bias can amplify the positive effects of grit on employability and life satisfaction for male teachers (Polat and İskender, 2018).

Conversely, females may face negative stereotypes that undermine their perseverance and goal-oriented efforts (Xu et al., 2023). Gender biases can result in women being perceived less favorably when exhibiting the same traits, thereby diminishing the positive impact of their grit on employability and life satisfaction. Despite possessing high levels of emotional resilience and grit, female teachers might not receive the same recognition and opportunities as their male counterparts, leading to a moderated effect where the benefits of resilience on life satisfaction through grit and employability are less pronounced for women (Brouskeli et al., 2018).

Based on the above revised literature, the following hypotheses are proposed:

Direct effects:

*H1*: Emotional Resilience (X) has a direct positive effect on Life Satisfaction (Y).

Indirect effects:

*H2*: Emotional Resilience (X) has an indirect effect on Life Satisfaction (Y) through Grit (M1).

*H3*: Emotional Resilience (X) has an indirect effect on Life Satisfaction (Y) through Employability (M2).

*H4*: Emotional Resilience (X) has an indirect effect on Life Satisfaction (Y) through the chain mediation of Grit (M1) and Employability (M2).

Moderated mediation effects:

*H5*: Gender (W) moderates the indirect effect of Emotional Resilience (X) on Life Satisfaction (Y) through Grit (M1).

*H6*: Gender (W) moderates the indirect effect of Emotional Resilience (X) on Life Satisfaction (Y) through Employability (M2).

*H7*: Gender (W) moderates the indirect effect of Emotional Resilience (X) on Life Satisfaction (Y) through the chain mediation of Grit (M1) and Employability (M2).

**Figure 1:**
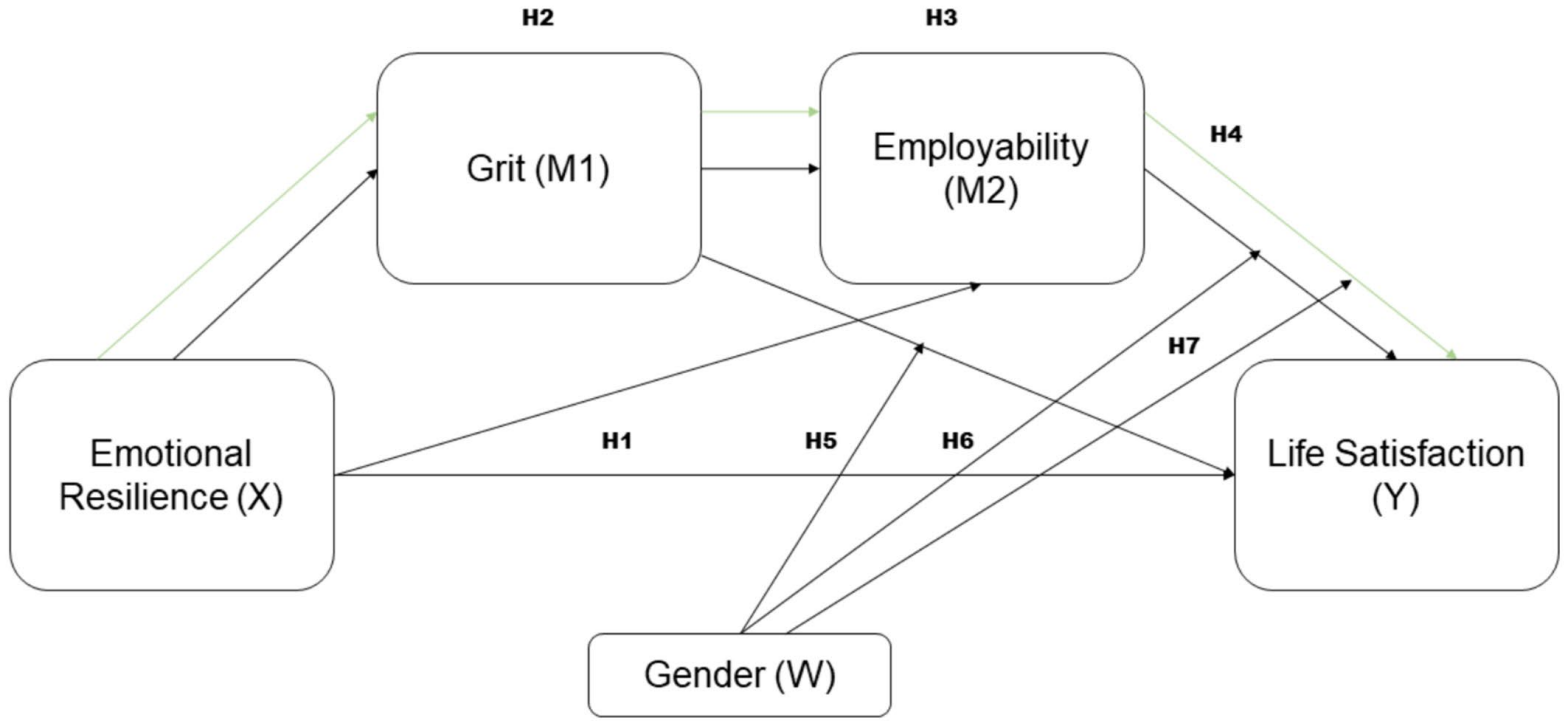
Process model 88 framework.

These hypotheses will guide the analysis to explore the complex relationships among Emotional Resilience, Grit, Employability, Gender, and Life Satisfaction as Figure 1 depicts through the PROCESS Model 88 framework.

## Methods

### Participants

The study comprised a total of 1,003 participants who are teachers of Chinese as a Foreign Language (CFL). The age of the participants ranged from 18 to 69 years, with a mean age of 37.18 years (SD = 11.47). Regarding gender, 56.4% were males. The participants had varying years of work experience with a mean of 8.63 years (SD = 9.45). The educational qualifications of the participants were bachelor’s degree (49.4%, *n* = 495), doctoral (Ph.D.) programs (20.3%, *n* = 204), and postgraduate (30.3%, *n* = 304). The participants were involved in different categories of professional education, including secondary education (6.3%, *n* = 63), courses of proficiency tests (19.3%, *n* = 194), higher education (24.9%, *n* = 250), adult education (40.8%, *n* = 409), and online education (8.7%, *n* = 87). The majority of participants were employed by private organizations (77.5%, *n* = 777), with the remaining participants working for public organizations (22.5%, *n* = 226). The distribution of participants based on the size of their organizations was as follows: organizations with 0–9 professors (21.2%, *n* = 213), organizations with 10–49 professors (20.6%, *n* = 207), organizations with 50–199 professors (10.8%, *n* = 108), and organizations with more than 200 professors (47.4%, *n* = 475).

### Procedure

This study was conducted in accordance with an approved protocol by the International Students Center, of the Beijing Youth Political College. Research was conducted following with the Declaration of Helsinki [18th World Medical Association (WMA) General Assembly, Helsinki, Finland, June 1964] and the latest amended (October 2013) in Fortaleza, Brazil, and in accordance with the local legislation about Research Involving Human Being [National Health Commission, No. 4 (2023) article 32]. The research protocol included several key steps to ensure the ethical and effective collection of data from participants. Prior to participation, all potential participants were provided with detailed information about the study’s objectives, procedures, potential risks, and benefits. They were informed that their participation was voluntary and that they could withdraw from the study at any time without any negative consequences. Participants gave their informed consent electronically before proceeding with the survey. To reach a broad and diverse group of CFL teachers, the survey was disseminated through various social networks. The primary platforms used for dissemination included WeChat, LinkedIn, and specialized forums such as the Chinese Language Teachers Association (CLTA) online community and relevant groups on Facebook. These channels were chosen for their popularity and relevance among CFL educators, ensuring a wide distribution of the survey and helping to gather data from a diverse sample of participants. Data were collected through a web-based survey named “CFL Teacher Professional Profile Survey,” during April–May 2024. *A priori* power analysis was conducted to determine the minimum sample size required for detecting statistically significant indirect and moderated effects within the model. Given the number of predictors, mediators, and interaction terms, a sample size exceeding 500 participants was deemed necessary to achieve sufficient statistical power. The chosen sample of 1,003 teachers exceeds this requirement, allowing for reliable estimates of both direct and indirect effects. This survey was designed to capture comprehensive information about the participants’ demographics, educational backgrounds, professional categories, type of firm, and organization size. The survey was structured to be user-friendly and accessible, allowing participants to complete it at their convenience. It included a mix of multiple-choice questions and open-ended questions to gather both quantitative and qualitative data.

### Instruments

*Emotional resiliency* was assessed using the Connor–Davidson Resilience Scale (CD-RISC-10), which is recognized for its strong psychometric properties. This scale evaluates an individual’s ability to overcome adversity and to recover from challenging situations.

The CD-RISC-10 consists of 10 items, each rated on a unidimensional 5-point Likert scale ranging from 0 (“Not true at all”) to 4 (“True nearly all the time”). This scale captures the frequency and intensity of resilience-related behaviors and attitudes. The overall score for each participant can range from 0 to 40, with higher scores indicating greater resilience. This scale is widely used in resilience research due to its reliability and validity in various Chinese subpopulations (Chen et al., 2022; Tu et al., 2023; Wang Y. et al., 2023; Wojujutari et al., 2024; Zhang et al., 2021). In the present study, the scale showed a Cronbach’s alpha of 0.789, indicating good internal consistency.

*Grit* was assessed using the Simple Chinese Version of the Grit Scale (GS-SC), which consists of 11 items. This scale evaluates two key components of grit: Consistency of Interest and Perseverance of Effort. Example items include “I often set a goal but later choose to pursue a different one” for Consistency of Interest and “I finish whatever I begin” for Perseverance of Effort. Participants responded to each item using a 5-point Likert scale, ranging from 1 (“Not like me at all”) to 5 (“Very much like me”). The reliability of the GS-SC was confirmed with a Cronbach’s alpha coefficient of 0.84 for the Consistency of Interest subscale and 0.72 for the Perseverance of Effort subscale. The overall scale demonstrated a Cronbach’s alpha of 0.79, indicating good internal consistency. Evidence for the construct validity of the GS-SC was provided by Ismail et al. (2023).

*Employability* was measured using the Self-Perceived Employability Scale, which consists of 11 items designed to assess perceived employability as a unitary construct. This scale, originally developed by Rothwell and Arnold (2007), was streamlined from a 16-item version to an 11-item version to enhance its practicality while maintaining its effectiveness (Rothwell et al., 2009). Participants responded to each item on a 5-point Likert scale, ranging from 1 (“Strongly disagree”) to 5 (“Strongly agree”). The Self-Perceived Employability Scale captures an individual’s perceptions of their employability, reflecting their confidence in their ability to secure and maintain employment. In the present study, the Self-Perceived Employability Scale underwent a back-translation procedure to ensure the accuracy and cultural equivalence of the translated items. A structured process was followed to maintain the integrity of the original scale while adapting it for the target population. Initially, the scale was translated from English to Chinese by a professional translator familiar with both languages and the subject matter. Next, a second independent translator, who was blinded to the original version, translated the Chinese version back into English. The back-translated version was then compared to the original English scale to identify any discrepancies in meaning or terminology. Minor adjustments were made to the Chinese version to resolve inconsistencies and ensure that the translated items retained the intended meanings of the original scale. This rigorous back-translation process helped enhance the linguistic and conceptual accuracy of the scale, ensuring that it was suitable for the study’s context and participants. In the present study, the scale showed a Cronbach’s alpha of 0.77.

*Life satisfaction* was assessed using the Satisfaction with Life Scale (SWLS) developed by Diener et al. (1985). This scale provides a global measure of life satisfaction and subjective wellbeing (SWB). It consists of five items, with responses measured on a 7-point Likert scale ranging from 1 (“Totally disagree”) to 7 (“Totally agree”). Higher scores indicate greater perceived life satisfaction. The Chinese version of the SWLS, which was utilized in this study, has demonstrated good validity and high internal consistency reliability, with a Cronbach’s alpha coefficient of 0.84. This makes it a reliable and valid tool for assessing life satisfaction among Chinese-speaking populations.

### Data analyses

Data analyses were conducted using IBM SPSS Statistics version 29.01.0 and the PROCESS macro developed by Andrew F. Hayes, specifically employing Model 88. First, descriptive statistics were computed for all variables to provide an overview of the sample characteristics. The internal consistency of the scales used (e.g., CD-RISC-10, GS-SC, Self-Perceived Employability Scale, SWLS) was assessed using Cronbach’s alpha coefficients. Pearson correlation coefficients were calculated to examine the relationships between key variables, such as emotional resiliency, grit, employability, and life satisfaction. To enhance the robustness of the mediation and moderation analyses, bootstrapping procedures were employed. A total of 5,000 bootstrap samples were generated to estimate the indirect effects and their confidence intervals. The bias-corrected bootstrap confidence intervals were used to determine the significance of the indirect effects. The lower level confidence interval (LLCI) and upper level confidence interval (ULCI) were reported for each analysis. An effect was considered significant if the 95% confidence interval did not include zero.

## Results

### Descriptive and correlational statistics

Table 1 presents the means, standard deviations, and Pearson correlation coefficients for the key variables in the study. These correlations suggest that higher levels of emotional resilience are associated with higher levels of grit, employability, and life satisfaction. Additionally, higher levels of grit are associated with higher levels of employability and life satisfaction. The strong correlation between employability and life satisfaction underscores the importance of employability as a predictor of overall life satisfaction.

**Table 1:** Descriptive statistics and Pearson correlations among study variables.

Variable	Mean	SD	1	2	3	4
1. Emotional resilience (X)	4.05	0.86	–			
2. Grit (M1)	3.98	0.66	0.322**	–		
3. Employability (M2)	4.08	0.58	0.414**	0.686**	–	
4. Life satisfaction (Y)	3.54	0.85	0.302**	0.477**	0.656**	–

**p < 0.01.

### Direct and mediating effects

The results of the regression analysis examining the relationship between Emotional Resilience (X) and Grit (M1) are summarized in Table 2. The model demonstrates a statistically significant relationship, with an F-statistic of 116.05 (*p* < 0.001). The R-squared value of 0.104 indicates that approximately 10.4% of the variance in Grit is explained by Emotional Resilience. The regression coefficients indicate that emotional resilience (X) has a positive and statistically significant effect on grit. The 95% confidence interval for the emotional resilience coefficient ranges from 0.203 to 0.294, suggesting that the true population parameter lies within this interval.

**Table 2:** Coefficients for emotional resilience predicting grit (M1) and employability (M2).

Outcome variables	Coeff	SE	t-value	p-value	LLCI	ULCI
Grit (M1)						
Constant	2.983	0.096	31.25	0.000	2.796	3.171
Emotional resilience (X)	0.248	0.023	10.77	0.000	0.203	0.294
Employability (M2)						
Constant	1.325	0.087	15.21	0.000	1.154	1.496
Emotional resilience (X)	0.146	0.016	9.22	0.000	0.115	0.177
Grit (M1)	0.543	0.021	26.44	0.000	0.503	0.583
Life satisfaction (Y)						
Constant	−0.009	0.449	−0.019	0.985	−0.889	0.872
Emotional resilience (X)	0.034	0.026	1.316	0.189	−0.017	0.085
Grit (M1)	0.323	0.128	2.514	0.012	0.071	0.574
Employability (M2)	0.535	0.146	3.663	0.0003	0.248	0.822
Gender	−0.323	0.298	−1.083	0.279	−0.907	0.262
Grit M1 × Gender	−0.182	0.085	−2.144	0.032	−0.349	−0.016
Employability M2 × Gender	0.248	0.097	2.565	0.011	0.058	0.437

The second part of the model, focusing on Employability (M2) as the outcome variable, showed that the model demonstrates a strong and statistically significant relationship between the predictors (emotional resilience and grit) and employability, with an F-statistic of 525.37 (*p* < 0.001). The R-squared value of 0.512 indicates that approximately 51.2% of the variance in employability is explained by emotional resilience and grit.

The regression coefficients indicate that both emotional resilience (X) and Grit (M1) have significant positive effects on employability. Specifically, emotional resilience has a coefficient of 0.146, and grit has a coefficient of 0.543.

Finally, the results of the mediation analysis examined the impact of emotional resilience on life satisfaction (Y) through both mediators, grit (M1) and employability (M2). The model demonstrates a statistically significant relationship between the predictors and life satisfaction. The R-squared value of 0.437 indicates that approximately 43.7% of the variance in life satisfaction is explained by the model.

The direct effect of emotional resilience on life satisfaction is positive but not statistically significant, with a 95% confidence interval ranging from −0.017 to 0.085. This suggests that, while emotional resilience tends to increase life satisfaction, the effect is not strong enough to be statistically significant on its own.

Grit has a positive and statistically significant direct effect on life satisfaction, with a 95% confidence interval ranging from 0.071 to 0.574. This indicates that individuals with higher levels of grit tend to report higher life satisfaction.

Employability also shows a positive and statistically significant direct effect on life satisfaction, with a 95% confidence interval ranging from 0.248 to 0.822. This suggests that individuals with higher employability tend to have higher life satisfaction.

The results indicate that both grit and employability mediate the relationship between emotional resilience and life satisfaction. Although the direct effect of emotional resilience on life satisfaction is not statistically significant, its impact is channeled through grit and employability, which both show significant positive effects on life satisfaction.

These findings underscore the importance of grit and employability as mediators. Emotional resilience contributes to higher grit and employability, which in turn lead to greater life satisfaction. This highlights the complex pathways through which emotional resilience influences overall wellbeing.

### Moderation of gender

**Figure 2:**
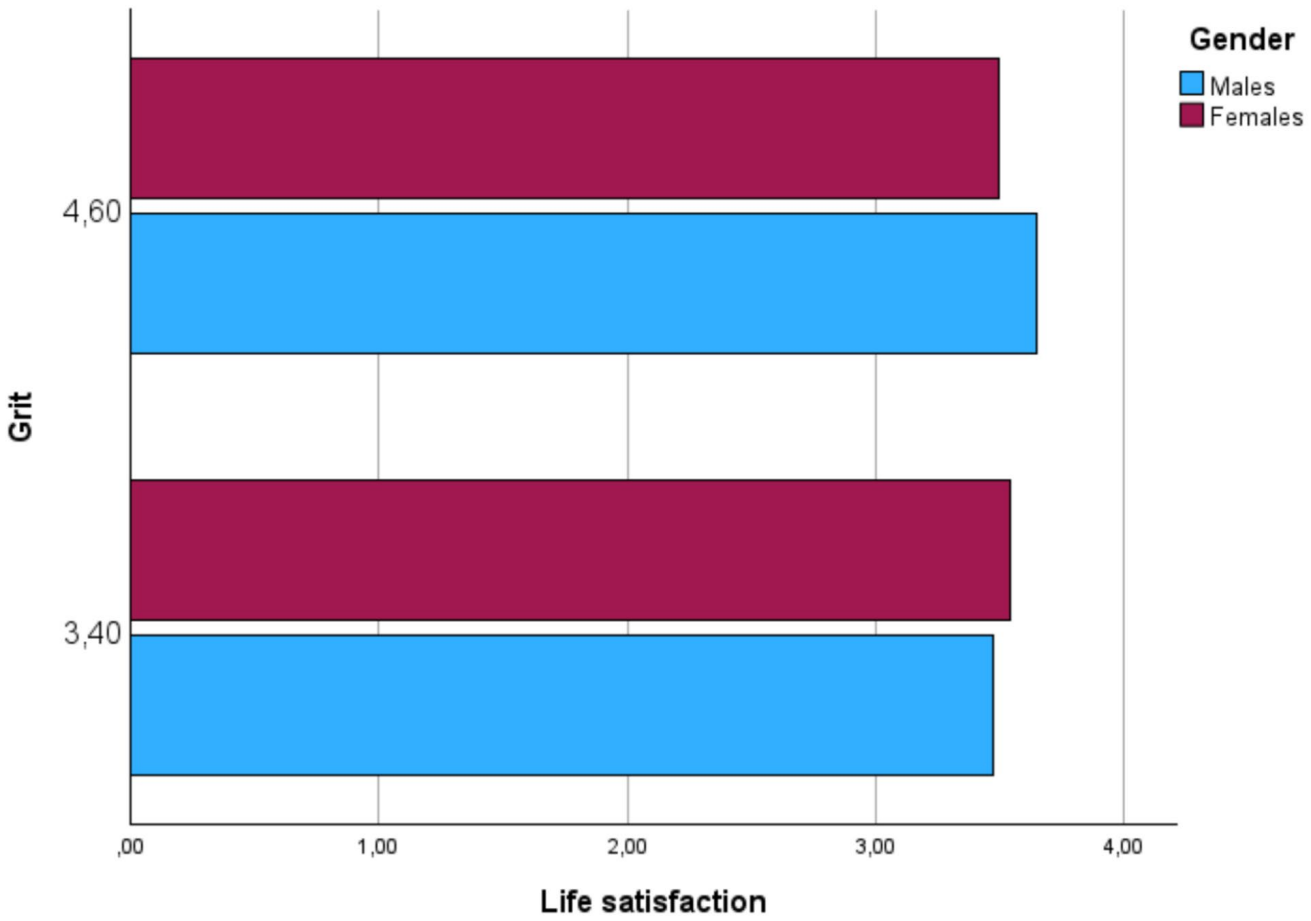
Scatterplot for the conditional effects of grit on life satisfaction.

**Figure 3:**
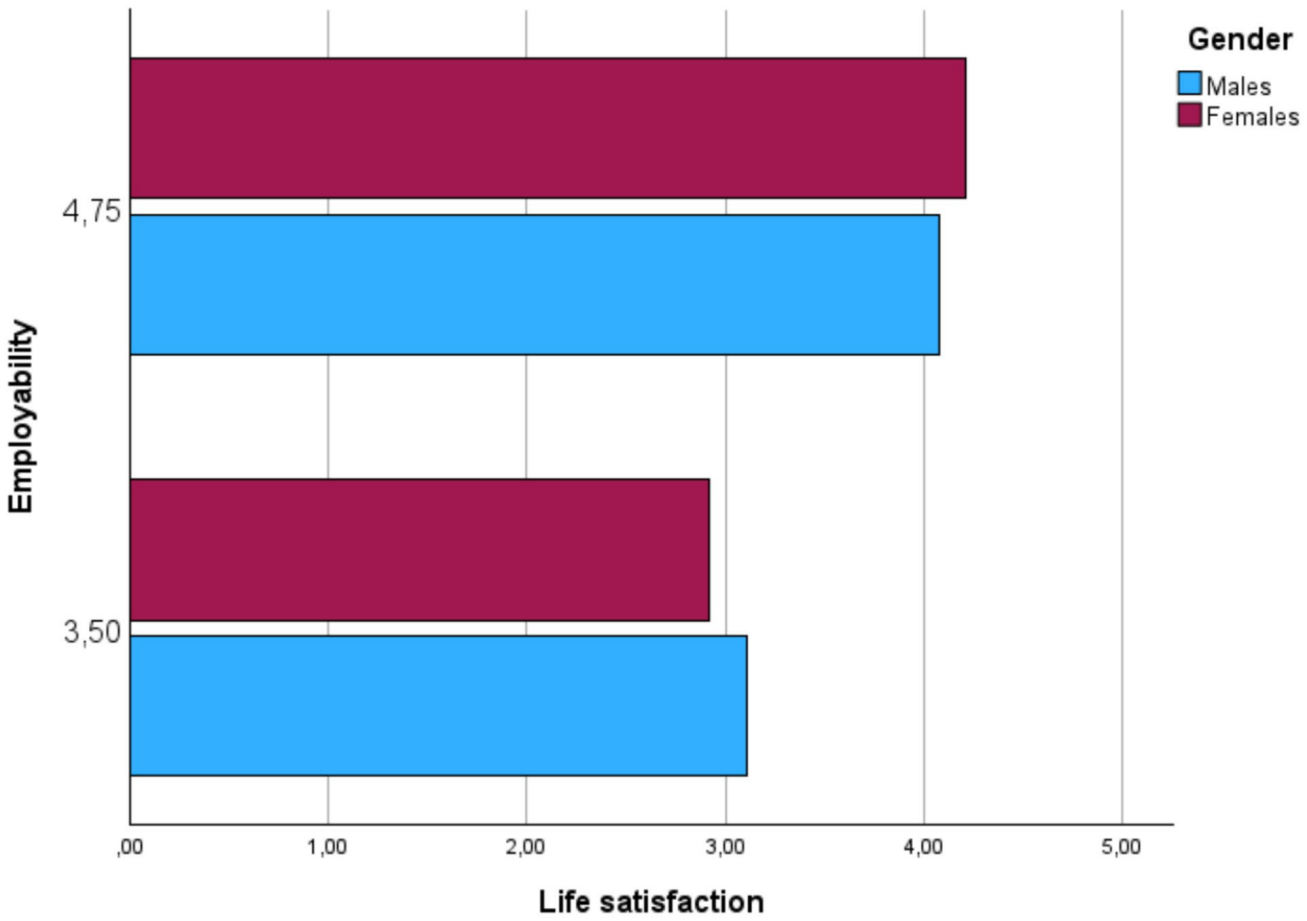
Scatterplot for the conditional effects of employability on life satisfaction.

As Table 2 showed, the regression analyses supported that, despite that Gender does not significantly predict Life Satisfaction, both interactions (Grit × Gender, and Employability × Gender) were significant. As Figures 2, 3 shown, Gender significantly moderates the direct relationships between Grit and Life satisfaction, on one hand, and Employability and Life Satisfaction, on the other hand.

The analysis for Hypothesis 5 examined whether gender moderates the indirect effect of emotional resilience on life satisfaction through grit. The interaction term between grit and gender was statistically significant, indicating that the relationship between grit and life satisfaction varies by gender. The conditional effects show that for males (Gender = 1), the indirect effect of emotional resilience on life satisfaction through grit is significant. However, for females (Gender = 2), this indirect effect is not significant. The index of moderated mediation is −0.0452, confirming that the indirect effect of emotional resilience on life satisfaction through grit is significantly moderated by gender.

For Hypothesis 6, the analysis investigated whether gender moderates the indirect effect of emotional resilience on life satisfaction through employability. The interaction term between employability and gender was statistically significant, indicating a gender difference in how employability affects life satisfaction. The conditional effects show that for males, the indirect effect of emotional resilience on life satisfaction through employability is significant. For females, this indirect effect is also significant but stronger. The index of moderated mediation is 0.0361, confirming that the indirect effect of emotional resilience on life satisfaction through employability is moderated by gender.

Hypothesis 7 examined whether gender moderates the chain mediation effect of emotional resilience on life satisfaction through both grit and employability. The conditional indirect effects show that for males, the indirect effect through the chain mediation of grit and employability is significant. For females, this indirect effect is also significant and stronger. The index of moderated mediation is 0.0334, indicating that the chain mediation effect of emotional resilience on life satisfaction through grit and employability is significantly moderated by gender.

These results suggest that gender plays a significant moderating role in the indirect effects of emotional resilience on life satisfaction through both grit and employability, as well as through the combined pathway of grit and employability.

These tables illustrate the moderated mediation effects, with gender significantly influencing the indirect pathways from emotional resilience to life satisfaction through both grit and employability, as well as through the combined mediation pathway of grit and employability. Specifically, the indirect effect of emotional resilience on life satisfaction through grit is significant for males but not for females. In contrast, the indirect effects through employability and through the chain mediation of grit and employability are significant for both genders, with stronger effects observed for females (Table 3).

**Table 3:** Conditional indirect effects of emotional resilience (X) on life satisfaction (Y).

	Effect	BootSE	BootLLCI	BootULCI
Outcome variable: life satisfaction (Y) through grit (M1)				
Males	0.035	0.014	0.007	0.064
Females	−0.010	0.018	−0.046	0.026
Outcome variable: life satisfaction (Y) through employability (M2)				
Males	0.114	0.016	0.084	0.146
Females	0.150	0.020	0.113	0.190
Outcome variable: life satisfaction (Y) through the chain mediation of grit (M1) and employability (M2)				
Males	0.106	0.016	0.075	0.139
Females	0.139	0.019	0.103	0.179

## Discussion

The findings of this study provide insight into the complex interplay between emotional resilience, grit, employability, and life satisfaction. Starting with the direct effects, Hypothesis 1 (H1) posited that emotional resilience (X) would have a direct positive effect on life satisfaction (Y). The results, however, indicate that while emotional resilience does have a positive coefficient, this effect is not statistically significant. This suggests that emotional resilience alone may not be a strong enough predictor of life satisfaction for CFL teachers, possibly due to the complex challenges they face in balancing teaching effectiveness with cultural promotion. The 95% confidence interval ranging from −0.017 to 0.085 supports this interpretation, indicating that the true effect could be negligible or slightly positive. Therefore, H1 is not supported by the data, highlighting the necessity of exploring other pathways through which emotional resilience might impact life satisfaction.

These findings partially align with the broader literature on resilience and wellbeing. Studies such as Liu et al. (2024), which emphasize how CFL teachers’ identity development in intercultural settings affects wellbeing, suggest that resilience may work indirectly rather than through a direct influence. Similarly, while Luthans et al. (2007) emphasized the strong direct effect of psychological capital (which includes resilience) on wellbeing and job performance, the non-significant direct effect in our study may be explained by differences in study populations or teaching contexts, particularly among CFL teachers. Nie (2023), who highlights technological and structural challenges faced by CFL teachers, suggests that resilience in this specific group may be insufficient by itself without additional mediators such as grit or employability.

Conversely, studies like Cohn et al. (2009) found a significant direct relationship between emotional resilience and life satisfaction. Their findings suggest that resilience can directly enhance life satisfaction, contrasting with our results. The differences may be attributed to the unique challenges CFL teachers face in managing diverse student populations and cross-cultural responsibilities, which could dilute the direct effect.

Hypothesis 2 (H2) proposed that emotional resilience would influence life satisfaction indirectly through grit (M1). The significant positive effect of emotional resilience on grit demonstrates that emotionally resilient individuals tend to have higher levels of grit. Furthermore, grit itself has a significant positive effect on life satisfaction, indicating that emotional resilience enhances life satisfaction indirectly by fostering greater grit, thus supporting H2. The R-squared value of 0.104 for the model predicting grit suggests that while emotional resilience is a significant predictor, other factors also contribute to grit. Studies like Fu (2025) confirm that grit plays a mediating role in various educational contexts by linking motivation, perseverance, and satisfaction. The role of grit in fostering long-term goals and emotional stability is also emphasized by Duckworth et al. (2007) and Eskreis-Winkler et al. (2014), aligning with our findings that grit is a crucial pathway connecting emotional resilience to life satisfaction. Hypothesis 3 (H3) suggested that employability (M2) would be another pathway through which emotional resilience affects life satisfaction. The data supports this hypothesis, as emotional resilience significantly predicts employability, which in turn positively influences life satisfaction. The model predicting employability has an R-squared value of 0.512, indicating that emotional resilience and grit explain a substantial portion of the variance in employability. This finding aligns with Huang (2024), who explored how foreign language teachers’ information literacy and professional adaptability contribute to their employability and overall satisfaction. Fugate et al. (2004) and Berntson et al. (2006) also found that employability enhances life satisfaction by providing individuals with a sense of security and fulfillment, supporting H3. In the context of CFL teachers, Liang and Apedoe (2025) highlight that employability factors, such as adapting to curriculum changes and language fluency development, are critical for success and wellbeing.

Hypothesis 4 (H4) posited a chain mediation effect involving both grit and employability. The results show that emotional resilience positively affects grit, which then enhances employability, and both grit and employability significantly improve life satisfaction. The chain mediation highlights how psychological traits and professional development work together to enhance wellbeing. This comprehensive pathway supports the idea that resilience must foster specific traits and professional competencies to influence life satisfaction, as suggested by studies like Xu et al. (2024), who examined teachers’ immediacy and clarity as mediators for reducing emotional stress in foreign language classrooms. Gender differences emerged as a significant factor in these pathways, as indicated in Hypotheses 5, 6, and 7. Hypothesis 5 proposed that gender moderates the indirect effect of emotional resilience on life satisfaction through grit. The results confirm that this pathway is significant for males but not for females, implying that grit plays a more critical role for males in achieving life satisfaction. This finding partially aligns with Diseth et al. (2014), who observed that grit had a stronger impact on academic success in males. Conversely, Clark and Malecki (2019) suggested that grit is equally important for both genders, contrasting with our results. The discrepancy might be explained by cultural expectations, as Yang et al. (2025) suggest that cultural and psychological capital can shape gender-specific outcomes in foreign language teaching.

Hypothesis 6 found that gender moderates the pathway from emotional resilience to life satisfaction through employability, with the effect being stronger for females. Employability’s significant impact on females’ life satisfaction may be due to its role in providing a sense of purpose and professional security (McQuaid and Lindsay, 2013; Wang H. et al., 2023). This finding also aligns with the work of Sun and Luo (2024), who emphasize the role of career stability in enhancing wellbeing, particularly among female educators.

Hypothesis 7 posited that gender moderates the chain mediation effect of emotional resilience on life satisfaction through both grit and employability. The results confirm that the combined effect of grit and employability is more pronounced for females, suggesting that interventions aimed at improving life satisfaction should be tailored differently for each gender. This gender-specific approach is supported by Tomkova et al. (2022), who emphasize the importance of gender-based differences in resilience outcomes. Conversely, studies like Karatepe and Olugbade (2009) found no significant gender differences, highlighting the need for further investigation into how cultural or contextual factors influence these outcomes.

Overall, the findings emphasize that emotional resilience exerts its influence on life satisfaction primarily through grit and employability, with gender-specific pathways adding complexity. For males, enhancing grit through resilience is key to improving life satisfaction, whereas for females, employability plays a more significant role. As Jiang (2025) and Li and Liu (2025) highlight, addressing contextual and gender-related factors in professional development programs is essential for promoting teacher wellbeing. Future research should further explore these gender-specific pathways in diverse teaching populations to provide comprehensive and tailored interventions.

### Limitations of the present study and suggestions for future research

While the study offers valuable insights into the professional profiles and psychological traits of teachers of CFL, several limitations must be acknowledged.

One significant limitation pertains to the sample representativeness. The study primarily reached participants through platforms such as WeChat, LinkedIn, and specific forums. These platforms may not be equally accessible or popular across all regions and cultural contexts, potentially introducing a bias in the sample. The reliance on these platforms might have excluded CFL teachers who are less engaged in digital or social media. Additionally, a significant majority of participants were employed by private organizations (77.5%). This overrepresentation may skew the findings, limiting the generalizability to public sector CFL teachers whose experiences and challenges might differ.

Another limitation relates to the nature of the survey. The study relied on self-reported data, which can be susceptible to social desirability bias and inaccurate self-assessment. Participants might have overestimated or underestimated their resilience, grit, employability, and life satisfaction due to personal biases or misunderstandings of the survey items. Furthermore, as a cross-sectional study, the research captures data at a single point in time. This design limits the ability to infer causality or understand changes in participants’ professional and psychological traits over time.

The measurement tools used in the study also present limitations. While the scales used (e.g., CD-RISC-10, GS-SC, Self-Perceived Employability Scale, SWLS) have demonstrated good reliability and validity in previous research, their application in this specific population may still have limitations. Cultural nuances and contextual factors unique to CFL teachers might not be fully captured by these scales. Moreover, although validated Chinese versions of the scales were used, subtle differences in language and cultural interpretation could affect the accuracy and consistency of responses.

To address these limitations and build on the findings of this study, future research should consider several recommendations. First, enhancing sample representativeness is crucial. Researchers should employ a more diverse range of recruitment methods to include CFL teachers who may not be active on social media platforms. Expanding the geographic and cultural scope of the sample will also help to provide a more comprehensive understanding of CFL teachers’ experiences across different contexts or educational institutions (Li and Yu, 2024), as well as the applicability of the current findings to other cultural groups or professional fields (Farinha et al., 2019; Kumar, 2024). Additionally, balancing the representation of private and public sector teachers will ensure the findings are more generalizable, given that public employees exhibit different working conditions in several areas (Lin et al., 2024).

Second, to mitigate the limitations of self-reported data, future studies could incorporate a mixed-methods approach, combining quantitative surveys with qualitative interviews. This would allow for a deeper exploration of participants’ experiences and reduce the potential biases associated with self-report measures. Longitudinal designs should also be considered to track changes in professional and psychological traits over time, providing insights into causality and developmental trends.

Third, refining the measurement tools to better capture the specific context of CFL teachers is essential, as the specificity of some features of Chinese (Yuen-han et al., 2024). Developing or adapting scales that consider cultural nuances and contextual factors unique to this population will improve the accuracy and relevance of the findings. Ensuring that these tools are thoroughly validated for use with CFL teachers will also enhance the reliability of the results.

### Practical implications for interventions

These results have practical implications for interventions aimed at improving the wellbeing of teachers of CFL. Given that emotional resilience does not have a direct significant effect on life satisfaction but exerts its influence through grit and employability, interventions should focus on these mediating factors. Specifically, programs designed to enhance grit and employability could be particularly effective in improving life satisfaction among CFL teachers.

To enhance grit, training programs that focus on developing perseverance and consistency of interest should be implemented. These could include workshops on goal setting, maintaining focus on long-term objectives, and strategies to overcome setbacks (Santos et al., 2022). Additionally, establishing mentorship programs where experienced teachers can guide less experienced colleagues in developing resilience and grit could foster a community of mutual support and shared learning (Mann et al., 2023).

Improving employability requires offering continuous professional development opportunities that enhance teachers’ skills and knowledge. This could include advanced teaching methodologies, language proficiency courses, and certifications that improve job prospects. Providing career counseling services can help teachers identify their strengths, explore career opportunities, and develop strategies to enhance their employability. Career counseling can also address job market trends and how to navigate them effectively. Facilitating networking events and platforms where teachers can connect with potential employers, colleagues, and professional organizations is another key strategy. Building a robust professional network can significantly enhance employability and career advancement.

Gender-specific interventions should be considered, as the study indicates different mediating factors for males and females. Interventions for male teachers should particularly emphasize the development of grit. Since grit is a more crucial mediator for males, programs that build perseverance and dedication to long-term goals will likely have a significant impact on their life satisfaction. For female teachers, interventions should focus more on employability. Given that employability has a stronger impact on life satisfaction for females, providing resources and support to enhance their career prospects will be particularly beneficial. This might include targeted professional development, leadership training, and opportunities to assume higher responsibilities within their institutions.

Developing comprehensive support programs that address both grit and employability simultaneously is essential. By fostering emotional resilience (Lee, 2023), enhancing perseverance, and improving career prospects, these programs can create a more holistic approach to boosting life satisfaction among CFL teachers. Longitudinal support systems that accompany teachers throughout different stages of their careers should also be considered. This could involve periodic training sessions, continuous mentorship, and regular career assessments to ensure sustained development and wellbeing (Zhang et al., 2024).

Context-specific strategies must be tailored to account for cultural and contextual differences among CFL teachers. Programs should be adaptable to various educational environments and responsive to the specific needs and challenges faced by teachers in different regions and institutional settings.

In summary, the study highlights the importance of focusing on grit and employability to enhance life satisfaction among CFL teachers. By implementing targeted interventions that address these mediating factors, educators can be better supported in their professional and personal development. Future research should continue to explore these pathways and develop tailored strategies that consider gender differences and cultural contexts, ultimately contributing to more effective and sustainable interventions for improving teachers’ wellbeing.

## Conclusion

To sum up, this study highlights the indirect pathways through which emotional resilience affects life satisfaction among CFL teachers, offering a more nuanced understanding of the underlying mechanisms. While the direct effect of emotional resilience on life satisfaction is not statistically significant, its influence is transmitted through its positive impact on grit and employability. These mediating factors are crucial in promoting long-term wellbeing, particularly in the context of CFL teachers who face complex professional and cultural challenges.

The novelty of this study lies in its emphasis on gender-specific differences in these pathways, an aspect that has received limited attention in prior research. The findings reveal that grit plays a more significant role for males, while employability has a stronger effect for females, suggesting that tailored interventions are needed to support both groups. This gender-based approach adds to the growing body of literature that calls for context-specific and targeted strategies in promoting teacher wellbeing.

In terms of contributions to current knowledge, this study extends existing research on resilience by demonstrating the importance of psychological traits and professional competencies as mediators. Unlike previous studies that have largely focused on the direct effects of resilience, this research emphasizes the complex interplay between resilience, grit, and employability, highlighting the importance of indirect pathways. The study also contributes to the broader understanding of CFL teachers’ wellbeing by addressing specific factors related to their unique teaching context, such as intercultural challenges and gender dynamics.

From a practical perspective, the findings suggest that interventions aimed at improving life satisfaction among CFL teachers should not only focus on building emotional resilience but also prioritize the development of grit and employability. For male teachers, programs designed to foster perseverance and long-term goal-setting (grit) may be particularly beneficial. For female teachers, initiatives that enhance employability through professional development and career advancement opportunities may have a stronger impact.

Future research should build on these findings by exploring additional mediating factors, such as job satisfaction and self-efficacy, and by examining how cultural and contextual differences influence the observed pathways. By doing so, a more comprehensive understanding of teacher wellbeing can be developed, ultimately contributing to improved support mechanisms for teachers in diverse educational contexts.

## Data Availability

The raw data supporting the conclusions of this article will be made available by the authors, without undue reservation.
